# Human Factors in Brain–Computer Interfaces: Boundaries, Key Issues, and Methodological Approaches

**DOI:** 10.3390/brainsci16101066

**Published:** 2026-10-03

**Authors:** Liu Yan, Tianwen Li, Lei Zhao, Anmin Gong, Hao Fang, Wuheng Zuo, Yunfa Fu

**Affiliations:** 1 Faculty of Information Engineering and Automation, Kunming University of Science and Technology, Kunming 650500, China; 2 Brain Cognition and Brain-Computer Intelligence Integration Group, Kunming University of Science and Technology, Kunming 650500, China; 3 Faculty of Science, Kunming University of Science and Technology, Kunming 650500, China; 4 School of Information Engineering, Engineering University of PAP, Xi’an 710086, China; gonganmincapf@163.com; 5 School of Art and Design, Wuhan Institute of Technology, Wuhan 430205, China; 6 School of Educational Science and Technology, Zhejiang University of Technology, Hangzhou 310023, China; whyzuo@163.com

**Keywords:** brain–computer interface human factors engineering, neurocognitive adaptation, brain–computer co-adaptation, closed-loop interaction validation, multi-dimensional human factors evaluation, scenario-based human factors design

## Abstract

The large-scale, real-world deployment of brain–computer interface (BCI) is confronted with human factors bottlenecks, including reliability and usability. While modern human factors engineering has encompassed domains such as cognitive ergonomics, workload, adaptive automation, and human-in-the-loop systems, its theories and methodologies have historically placed their main emphasis on overt behavior and peripheral physiological measures, rather than on central neural activity as a primary control input. For the emerging interaction paradigm that takes central nervous activity as direct input and emphasizes long-term brain–computer co-adaptation, there remains a shortage of targeted conceptual and methodological frameworks. As a conceptual review, this paper discusses the necessity and disciplinary foundations for developing Human Factors Engineering for BCIs as an emerging interdisciplinary research direction. First, grounded in the defining features of BCI interaction—such as the implicit nature of input and brain–computer co-adaptation—it delineates the research boundaries and summarizes the core research objects as user neurocognitive characteristics, brain–computer collaborative adaptation mechanisms, multimodal feedback regulation, and contextual adaptation patterns. Second, it identifies three core scientific questions: individual differences and dynamic adaptation, human factors optimization and fault tolerance, and contextual generalizability principles, with ethics, privacy, and user rights and interests as an overarching dimension. Furthermore, it constructs a methodological system consisting of multimodal measurement and modeling, closed-loop validation, and multidimensional evaluation, and examines the supporting role of artificial intelligence approaches within this framework. Centered on the collaborative adaptation across neural, cognitive, and system levels, Human Factors Engineering for BCIs represents a deepening of the human-centered design philosophy. It provides theoretical reference for user-friendly BCI design and extends the research boundaries of human factors engineering.

## 1. Introduction

### 1.1. Research Background

With the rapid advancement of neural engineering and artificial intelligence technologies, brain–computer interfaces (BCIs) are accelerating their transition from laboratory research to diverse application scenarios, including medical rehabilitation, intelligent control, and immersive interaction [1,2,3]. By acquiring and decoding central neural activity signals such as electroencephalography (EEG), functional near-infrared spectroscopy (fNIRS), and functional magnetic resonance imaging (fMRI), BCIs bypass peripheral neural and muscular pathways and directly translate human intentions into control commands for external devices, thereby offering a novel paradigm for human–computer interaction [4,5,6].

However, the large-scale deployment of BCI technology still faces multiple bottlenecks. Current research and industrial practices largely focus on optimizing decoding algorithms and improving hardware performance. Nevertheless, issues such as significant inter-individual neural variability, high temporal volatility of brain signals, excessive cognitive load during interaction, and insufficient long-term system adaptivity have not been systematically addressed. Consequently, the reliability, usability, and user acceptance of BCI systems remain insufficient for sustained real-world deployment [7,8]. As decoding algorithms and hardware continue to advance, the problem of human–brain–system fit is increasingly becoming a critical factor that constrains the long-term application and large-scale adoption of BCIs.

Over decades of development, modern Human Factors Engineering (HFE) has established a relatively complete framework covering physical ergonomics, cognitive ergonomics, and organizational ergonomics. Workload, situational awareness, human–machine trust, adaptive automation, human-in-the-loop supervisory control, and neurophysiological measurements have all become well-established research topics within this field [9,10]. Nevertheless, in much of this work the human is modeled mainly through overt behavior and peripheral physiological activities, while implicit states such as attention, workload, and affect are more often treated as states to be monitored or mitigated than as signals that simultaneously drive and are shaped by the interaction. Human intentions are translated into limb movements or vocal outputs via neuromuscular pathways, based on which the system perceives and responds to human operations. BCI offers an alternative interaction pathway: it directly acquires and decodes central neural activity into control commands, bypassing peripheral nerves and muscles [11,12,13,14]. Within this pathway, the generation of signals, origins of errors, and modulation of user states through feedback cannot be fully explained by classic human–machine interaction models. Interaction performance is no longer determined solely by overt behaviors and peripheral physiological parameters; instead, it is heavily influenced by fluctuations in central neural signals, changes in implicit cognitive states, and neuroplastic processes [8,11,12,13,14]. These implicit and dynamic variables are generally not treated as interaction inputs in conventional human factors research, requiring dedicated concepts and methodologies for characterization.

Distinct differences also exist in their design orientations. Classic human factors approaches have largely been developed for task workflows in which the operator and the system are allocated relatively stable roles, although adaptive automation and dynamic function allocation have long addressed temporal variation within a task. By contrast, BCI represents a typical dynamic closed-loop human-in-the-loop system. Users’ brain states change in real time and neural functions continuously remodel with training, shifting the design focus from one-off static matching to sustained online regulation and co-adaptation [15,16,17,18,19]. It is important to emphasize that this distinction does not negate existing HFE. Instead, it indicates that direct adoption of existing frameworks suffers from poor adaptability when the interaction pathway, research subjects, and temporal scales change. Targeted theories and methods therefore need to be developed on this foundation.

Therefore, it is imperative to integrate neuroscience, BCI technology, and human–computer interaction design approaches into the existing human factors engineering framework, and to establish BCI human factors engineering as a new interdisciplinary research direction specifically dedicated to brain–computer interaction scenarios. Centered on users’ central nervous system characteristics and cognitive states, this research direction inherits the core ethos of human-centered design while further expanding the research boundaries and methodological scope of human factors engineering. Its core objective is to break through the implementation bottlenecks of BCI technology from a human factors perspective, and to advance BCI systems from mere technical viability to genuine user usability.

**Figure 1:**
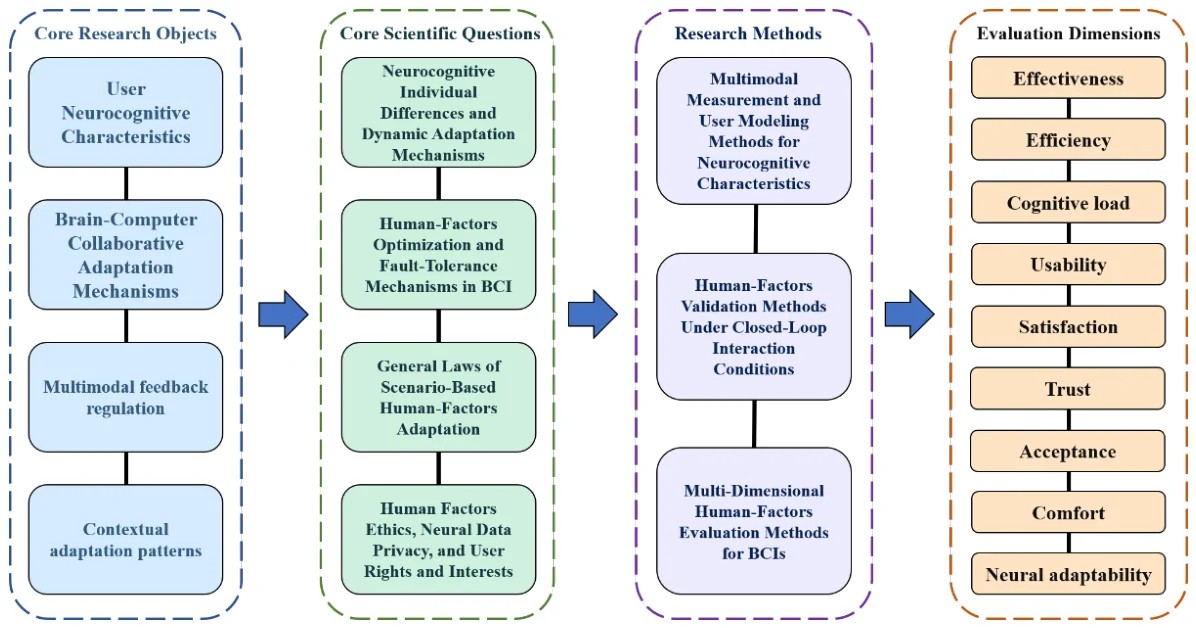
Overall framework of BCI human factors engineering research.

Focusing on the research boundaries of HFE for BCIs, this paper summarizes the core scientific questions in this field and constructs a BCI-specific methodological system and evaluation framework, as illustrated in Figure 1. It further examines the positioning and underlying rationale of this area as an emerging interdisciplinary research direction within HFE, aiming to provide a theoretical foundation and methodological pathway for the human-centered design of BCI systems while expanding the research scope of HFE.

### 1.2. Literature Review Methodology

This paper is a conceptual review based on a purposive, theory-driven literature search rather than an exhaustive systematic search. Rather than providing a reproducible statistical summary of all literature on a specific empirical issue, this paper aims to integrate and conceptualize studies scattered across fields such as BCI engineering, HFE, neuroergonomics, and human–computer interaction, centering on research boundaries, core scientific questions, and methodological systems. By delineating core concepts, clarifying disciplinary boundaries, and synthesizing research objects, scientific questions, and methodological categories, this paper proposes a structured analytical framework. Accordingly, no claim of exhaustive coverage is made.

For the literature base, this study takes Web of Science, PubMed, IEEE Xplore, Scopus and China National Knowledge Infrastructure (CNKI) as the main retrieval databases. The search terms are organized into four concept groups:

(1) BCI: “brain–computer interface” or “brain computer interface” or “BCI”;
(2) Human factors/ergonomics: “human factors” or “ergonomics” or “neuroergonomics”;
(3) HCI/usability/user experience: “human–computer interaction” or “usability” or “user experience”;
(4) co-adaptation: “co-adaptation” or “coadaptation”.

The fourth concept group requires explanation. Brain–computer co-adaptation is the core feature that distinguishes BCI interaction from conventional human–computer interaction; using it as an anchor efficiently locates literature focused on the dynamic BCI interaction process rather than on hardware design or algorithm performance alone. This criterion is used to focus the scope of the review; it is not intended to define the boundaries of the field, nor does it imply that human factors research related to BCIs necessarily uses this terminology.

To be included, a record had to contain at least one term from each of the four concept groups; within each group, any listed term was sufficient. The search was conducted in titles, abstracts, and keywords. The time scope primarily covers publications from 2010 to 2025, supplemented by seminal early works in the field as appropriate. The inclusion criteria are defined as follows: journal articles, reviews, and high-quality conference papers addressing BCI user characteristics, human–computer interaction, usability and user experience, co-adaptation, feedback design, evaluation methods, or human factors issues in specific application scenarios. Literature focusing on pure hardware design or decoding algorithm performance comparison without involving user or human factors analysis is only cited when describing the technical background.

It should be noted that the intersection search served only as an entry point for the initial screening of the literature, rather than as the boundary of the final corpus. During the screening and thematic clustering stage, the corpus was further supplemented through three routes: first, backward and forward citation chasing from the included reviews and seminal contributions; second, manual screening and supplementation based on the reference lists of highly cited papers and key reviews in the field; and third, targeted checks of whether important studies addressing BCI usability, cognitive load, trust, privacy, and calibration that do not use the term “co-adaptation” had been omitted, with such studies included where relevant. Records identified through these routes were screened against the same inclusion criteria as the retrieved records and were treated identically during thematic clustering.

Because the intersection search prioritizes theoretical specificity, BCI human factors studies that do not use the term “co-adaptation” may not have been covered by that route alone; backward and forward citation chasing, supplementation with highly cited works, and manual screening were therefore used to compensate for this limitation. The corpus should thus be regarded as a purposive sample supporting conceptual synthesis rather than an exhaustive collection of the literature.

For evidence synthesis, this paper first conducts thematic clustering of the literature and then performs theoretical deduction grounded in the hierarchical logic of BCI closed-loop interaction, so as to synthesize the core research objects, scientific questions, and methodological categories. The specific derivation procedure is described below.

Stage 1: open coding and extraction of initial themes. Each of the initially retrieved papers was coded in terms of the user-related variables examined, the element of the closed loop involved, the human factors problem reported, the measurement and evaluation methods adopted, and the application scenario; on this basis, more than 20 initial research themes were extracted, including “individual neural differences”, “cognitive load measurement”, “multimodal feedback”, “fault-tolerant design”, “usability evaluation”, “scenario-based application”, and “ethics and privacy”.

Stage 2: hierarchical aggregation and category induction. Based on the system logic of BCI closed-loop interaction, the initial themes were hierarchically grouped to form four core research objects: “user neurocognitive characteristics” corresponding to the user input stage, “brain–computer collaborative adaptation mechanisms” corresponding to collaborative interaction, “multimodal feedback regulation mechanisms” corresponding to feedback regulation, and “scenario-based human factors adaptation principles” corresponding to scenario deployment.

Stage 3: mapping of questions and methods. For each category of research object, the corresponding core scientific questions were identified, and matching research methods and evaluation dimensions were assigned, ultimately consolidating into an overall framework of research boundaries, core questions, and methodological systems. Ethics and user rights and interests, which are attached to all four research objects rather than to a single element of the loop, were treated as a cross-cutting dimension.

These three stages constitute the pathway from the primary literature to the overall framework. It should be noted that this derivation is an interpretive, theory-driven conceptualization rather than an algorithmic or statistical induction; the proposed framework is an organizing scheme open to further testing and revision, and its value awaits empirical validation.

## 2. Research Scope of BCI Human Factors Engineering

To delineate the research domain and disciplinary positioning of BCI human factors engineering, this section elaborates its research scope from three perspectives: the human factors characteristics of BCI interaction, the core research objects, and the relationships with related fields.

### 2.1. Human Factors Uniqueness of BCI Interaction

The architecture and operational logic of BCI systems determine their distinct human factors attributes. A BCI system typically comprises seven constituent elements, with the user and their brain consistently occupying the central position in the interaction loop. The generation, acquisition, decoding, and feedback of brain signals are intrinsically coupled with the user’s cognitive and physiological states. The specific components are listed in Table 1 [20,21].

From a human factors perspective, the above architecture reveals that BCI interaction no longer presents the observable and decomposable behavioral sequences typical of conventional peripheral operations, but rather a set of human factors characteristics directly rooted in central neural activity, as summarized in Table 2. These characteristics fundamentally alter the human–machine relationship in BCIs and help explain why existing human factors theories and methods do not fully capture certain BCI-specific interaction mechanisms.

It should also be noted that the aforementioned human factors characteristics carry different weights across different types of BCIs. In terms of signal acquisition modalities, the human factors challenges of invasive BCIs are mainly concentrated on surgical safety, long-term implantation stability, neural tissue compatibility, and long-term psychological adaptation of implant recipients. For non-invasive BCIs, by contrast, more prominent issues include wear comfort, burden of prolonged wear, signal anti-interference capability, and the cost of repeated calibration.

In terms of interaction paradigms, active BCIs rely more heavily on users’ cognitive strategies and training effort; reactive BCIs (e.g., P300, SSVEP) place greater focus on attentional resource allocation and stimulus-induced fatigue; whereas passive BCIs focus more on the ecological validity of unobtrusive monitoring, the robustness of state recognition, and user acceptance of implicit sensing.

The characteristic framework presented in this paper targets the common issues shared by all types of BCIs. In specific applications, the priority of human factors concerns should be determined in light of the BCI type.

Human–computer interaction research has long investigated implicit and physiological input, including gaze and physiological computing. Nevertheless, the characteristics of BCI interaction collectively manifest a tightly coupled interplay among neural, cognitive, and systemic factors. Consequently, the human factors issues in BCI interaction cannot be fully addressed by static behavioral matching alone, but extend into a dynamic process of neural adaptation [17,19]. This implies that BCI human factors engineering cannot rely solely on conventional human–computer interaction optimization; instead, it should more explicitly incorporate neural state dynamics, brain–computer co-adaptation principles, and personalized design under long-term usage scenarios as core considerations, thereby extending existing HFE to address BCI-specific interaction mechanisms.

### 2.2. Core Research Objects of BCI Human Factors Engineering

Based on the fundamental structure of the closed-loop BCI interaction system and its human factors characteristics, this paper summarizes the core research objects of BCI human factors engineering into four interrelated dimensions: users’ neurocognitive characteristics, brain–computer co-adaptive mechanisms, multimodal feedback regulation mechanisms, and scenario-based human factors adaptation principles. Together, these four dimensions delineate the primary research scope and theoretical domain of BCI human factors engineering.

Within the research framework of BCI human factors engineering, users’ neurocognitive characteristics constitute the most fundamental research foundation and serve as the essential prerequisite for personalized BCIs. While traditional HFE has developed extensive theories and methods for behavioral capabilities and peripheral physiological traits, these approaches do not fully capture certain BCI-specific interaction mechanisms at the central neural level. BCI human factors engineering therefore extends its research boundary to the central neural level. The core concerns in this dimension can be classified into three categories: individual differences in neural activity patterns and cognitive abilities, and how such differences affect system performance; state fluctuations such as fatigue, attention, and emotion, which directly induce dynamic changes and non-stationarity in brain signals; and, importantly, the functional reorganization and adaptation mechanisms driven by neuroplasticity during long-term usage. Collectively, these factors determine that BCI systems must meet the requirements of dynamic adaptation across individuals, states, and time, thereby providing a neurocognitive basis for the design of personalized BCIs [22,23,24].

The brain–computer co-adaptive mechanism is another core research focus in this field, centering on elucidating the dynamic coupling process among user intent generation, neural encoding, algorithmic decoding, and system responses. The inherent uncertainties of the BCI interaction pathway—such as low signal-to-noise ratio, decoding latency, and erroneous recognition—necessitate investigations into the interactions among neural states, cognitive load, and system performance. Furthermore, it is equally important to examine how system feedback reciprocally modulates users’ cognitive strategies, trust-building processes, and operational behaviors, ultimately distilling the fundamental principles of brain–computer co-adaptive coordination [19,25,26,27].

Multimodal feedback regulation constitutes a vital component of closed-loop BCI interaction and represents a distinguishing human factors research emphasis compared with conventional human–computer interaction. Owing to the highly implicit nature of brain-controlled operations, feedback design directly affects users’ learning efficiency, cognitive load, and overall user experience [28,29]. This research direction primarily investigates the effects of different feedback modalities and their combinations on user perception, cognitive processing, and task performance, and further explores the underlying mechanisms through which neurofeedback contributes to brain activity regulation, skill acquisition, and closed-loop adaptation processes, thereby providing theoretical support for the design of efficient, low-load brain–computer interaction.

At the design level, the organization of multimodal feedback should form a unified mechanism centered on feedback timing, channel allocation, information redundancy, error handling, and the synergy of neurofeedback. Feedback timing must match the rhythm of users’ cognitive processing and action correction. Excessively long delays may impair operational continuity and sense of agency, while excessively short or overly frequent feedback may increase information interference and cognitive load. Accordingly, feedback delay and presentation frequency should be dynamically adjusted according to task characteristics and user states [30,31]. The configuration of feedback channels must balance task requirements and cognitive resource allocation. For motor control tasks, tactile or proprioceptive feedback can be adopted to reduce visual resource occupation; for attention and rhythm regulation tasks, auditory or visual channels can be utilized for information transmission, thereby reducing resource competition between task operation and feedback [32].

Multi-channel information should also establish appropriate redundancy and complementarity. Redundant coding of critical states and error information can improve discrimination capability under weak signal conditions, while semantic consistency across different channels must be maintained to avoid additional interference caused by feedback conflicts. Error feedback should support error identification and behavior correction, and control the intensity and duration of negative information to avoid excessive frustration or attentional fixation. Combined with the detection of users’ implicit error awareness via error-related potentials (ErrP), corresponding correction mechanisms can be activated before overt behaviors emerge [33,34]. The synergy between neurofeedback and external feedback modalities such as visual, auditory, and tactile feedback can further establish closed-loop associations among brain states, behavioral performance, and feedback information, facilitating the formation of effective cognitive strategies, human–machine co-adaptation, and neuroplasticity, while the additional cognitive load imposed by neurofeedback must be controlled. Existing studies have shown that virtual reality, auditory induction, and multimodal feedback can influence the speed of co-adaptation learning and skill retention [35,36,37].

Finally, at the applied level, attention must also be directed toward scenario-based adaptation principles—that is, the human factors requirements and design guidelines for BCI systems under varying task environments and application conditions. Different scenarios exhibit divergent task complexity, target user profiles, and environmental constraints, which in turn impose distinct demands on system comfort, real-time performance, safety, and reliability [38,39]. It is therefore necessary to construct context-specific human factors design frameworks that investigate the dynamic matching logic among technological features, user needs, and environmental conditions, and to synthesize generalizable scenario-based adaptation principles, so as to genuinely facilitate the transition of BCI systems from laboratory research to practical deployment.

The above four research dimensions collectively constitute the core research objects and theoretical boundaries of BCI human factors engineering. From a content perspective, this field not only inherits the fundamental human-centered philosophy of existing HFE, but also extends the research dimensions from behavioral and peripheral physiological levels to neural-cognitive and BCI closed-loop interaction levels, thereby forming a human factors research framework that integrates neuroscientific characteristics with BCI attributes. This expansion of research objects not only reflects the deepening impact of BCI technology on traditional human–computer interaction paradigms, but also provides an essential theoretical foundation for constructing a human factors research system tailored to BCIs.

### 2.3. Boundary Delineation with Related Disciplines

HFE for BCIs is closely related to traditional HFE, cognitive ergonomics, neuroergonomics, human–computer interaction, BCI engineering technology, user-centered BCI research, and neuroadaptive technology. There is clear overlap in their research objects and methodologies, and they are not isolated from one another. Table 3 sorts out the relationships between relevant disciplines and HFE for BCIs.

Compared with existing HFE, BCI human factors engineering inherits the fundamental human-centered philosophy, but extends its research scope to neurocognitive states, brain–computer co-adaptation, and long-term dynamic adaptation processes, rather than being confined to interaction optimization at the level of behavioral performance and peripheral physiological responses [46].

Compared with BCI engineering technology, BCI human factors engineering places greater emphasis on the overall human–system interaction efficacy and user experience, rather than solely on improving signal decoding and control performance [49]. Its research focuses include cognitive load, user satisfaction, long-term usage intention, and personalized adaptation. The two fields complement each other at the levels of technical implementation and practical deployment.

Its relationship with human–computer interaction, usability engineering, and user-centered BCI design is mainly reflected in overlaps at the methodological and application levels. Human–computer interaction and usability engineering have developed mature methodologies for task analysis, interaction design, usability testing, and user experience evaluation, while user-centered BCI design further emphasizes end-user needs and user participation in the design process [46,50,51]. These methods provide an important design and evaluation foundation for HFE for BCIs. In turn, HFE for BCIs incorporates factors such as neural states, implicit cognitive load, brain–computer adaptation, and long-term co-adaptation into its evaluation framework, enabling traditional usability evaluation dominated by behavioral and subjective reports to be further extended to the neural level.

In summary, BCI human factors engineering is grounded in the human factors issues arising from brain–computer interaction, and synthesizes relevant achievements from both traditional human factors engineering and BCI engineering technology, thereby forming a research perspective oriented toward long-term system adaptation and user experience optimization in brain–computer systems.

## 3. Core Scientific Questions of BCI Human Factors Engineering

BCI directly employs brain activity as the interaction medium, with ongoing dynamic interactions among user states, system feedback, and application environments. Consequently, the human factors issues in brain–computer interaction extend beyond behavioral optimization in traditional human–machine systems, covering neurocognitive variability, brain–computer synergy, and long-term scenario adaptation. On this basis, this paper synthesizes the core scientific questions of BCI human factors engineering into three major categories: neurocognitive individual differences and dynamic adaptation mechanisms, human factors optimization and fault-tolerance mechanisms in brain–computer interaction, and general laws of scenario-based human factors adaptation.

### 3.1. Neurocognitive Individual Differences and Dynamic Adaptation Mechanisms

Centered on individual differences and dynamic changes in users’ neurocognitive characteristics, a core scientific question emerges: How do individual neural activity patterns, cognitive abilities, and brain-state fluctuations affect BCI interaction efficacy, and how can the system achieve continuous adaptation across individuals, over time, and across varying states?

From a research-content perspective, this scientific question primarily involves three interrelated dimensions: individual neural differences, dynamic state changes, and long-term brain–computer co-adaptation.

Significant inter-individual variability exists in neural activity patterns, cognitive abilities, and information-processing strategies, making it difficult for a unified interaction paradigm and decoding strategy to satisfy the needs of different users [52,53,54]. Establishing user models based on individual neural characteristics, and achieving personalized adaptation of training protocols, interaction modes, and system parameters, constitutes a critical prerequisite for improving BCI usability and user experience.

The influence of individual differences on BCI reliability and usability is highly paradigm-specific and brain region-specific. For motor imagery (MI) BCIs, the intensity, spatial distribution, and controllability of mu rhythm (approximately 8–13 Hz) event-related desynchronization (ERD) and beta rhythm (approximately 13–30 Hz) event-related synchronization (ERS)—elicited by users in the primary somatosensory cortex and supplementary motor area—directly determine the separability of imagined movements and decoding accuracy [55,56]. A significant proportion of users struggle to produce stable, separable sensorimotor rhythms, a phenomenon known as “BCI illiteracy” or insufficient BCI competence.

For BCIs based on event-related potentials such as P300, the amplitude and latency of the parietally distributed P3b component are shaped by attention allocation and working memory updating, and functional variations in the front-parietal attention network represent a key source of individual performance variability [57]. For steady-state visual evoked potential (SSVEP) BCIs, individual differences stem primarily from the frequency-following response properties of the occipital visual cortex to flickering stimuli, susceptibility to visual fatigue, and strategies for attentional resource regulation [58,59].

This means that personalized adaptation cannot be limited to the behavioral level. Instead, neural markers corresponding to the core brain systems that underpin specific paradigms should be measured, as a basis for user stratification and paradigm selection.

Beyond stable individual differences, users’ cognitive states also exhibit marked temporal dynamics. Factors such as cognitive load, attentional level, and fatigue continuously influence brain-signal characteristics and interaction performance. Under high cognitive load, limited attentional resources and working-memory capacity may reduce the stability of brain signals and increase the risk of erroneous operations [60]. Therefore, how to perceive user state changes in real time and establish dynamic response mechanisms is a key issue for achieving stable brain–computer interaction. In recent years, the development of multimodal state sensing and user-modeling approaches has provided new technical foundations for cognitive-state monitoring and adaptive regulation.

When the time scale is further extended, the brain–computer interaction process itself exhibits continuously evolving characteristics. Learning effects, neuroplasticity, and fatigue accumulation may alter users’ brain activity patterns and further affect system performance and interaction experience [61]. Consequently, how to construct brain–computer systems with continuous learning capabilities and long-term adaptation capacities, so as to achieve stable synergy across time and across states, represents a major challenge for BCI human factors engineering. Meanwhile, the adaptation requirements of users across different ages, health statuses, and functional backgrounds—such as older adults, patients with stroke, and individuals with neuromotor disorders—should also be incorporated into a unified human factors design framework [62,63].

Given that human factors research requires the integration of information across three levels—neural, cognitive, and behavioral—data collected from the same participant on different days may fluctuate markedly due to sleep, mood, fatigue, and electrode status [55,64,65]. Maintaining result consistency thus becomes a methodological challenge that must be addressed, and in practice it is typically controlled at three levels.

At the measurement level, standardized resting-state and task baselines are collected prior to each experiment. Task features are normalized against each participant’s daily baseline, and electrode placement, impedance thresholds, ambient lighting, and task duration are fixed to reduce drift in acquisition conditions [66].

At the modeling level, adaptive classifiers with online updating are adopted to continuously adjust models in response to changes in data distribution. Techniques including transfer learning, domain adaptation, and user stratification (metric learning) are applied to alleviate the limitations of single-day calibration by drawing on historical session data and data from other users. Meanwhile, peripheral physiological and behavioral features such as eye movements, heart rate, and electrodermal activity are fused to cross-correct state fluctuations in brain signals [67].

At the experimental design level, longitudinal baselines of individual characteristics are established through multi-session, cross-day longitudinal tracking. This approach distinguishes relatively stable trait differences from transient state fluctuations, and allows for reporting of cross-session reliability and performance retention [68].

It should be acknowledged that non-stationarity is an inherent property of brain signals and cannot be completely eliminated. The practical goal is to constrain fluctuations within an acceptable range through the aforementioned measures, and to explicitly report the cross-session generalization ability of models in evaluations.

The effective operation of BCI systems depends not only on signal-decoding capabilities, but more critically on the systematic understanding and dynamic adaptation to users’ neurocognitive characteristics and their patterns of change. The human factors modeling framework built around individual differences, state variations, and long-term co-adaptation further leads to higher-level human factors issues such as feedback design, fault-tolerance mechanisms, and user trust formation.

### 3.2. Human Factors Optimization and Fault-Tolerance Mechanisms in BCI Interaction

Beyond individual neurocognitive differences, the inherent uncertainties of the brain–computer interaction process itself constitute another important set of issues that BCI human factors engineering must address. Decoding errors, response latency, and even control deviations are inevitable in brain-signal decoding. How users interpret system behavior, form reasonable expectations, and maintain stable coordination over prolonged use remains a persistent challenge in BCI design [69]. Surrounding this issue, three interrelated key questions emerge: how users gradually adapt to brain-controlled interaction, how they build trust in the BCI system, and how the BCI system can implement effective fault tolerance.

For many users, adapting to a BCI is inherently a continuous learning process [70]. Given that decoding errors cannot be entirely eliminated, users are not always able to clearly discern whether a problem originates from their own intent expression or from system misinterpretation. Over time, this uncertainty may impede the formation of stable control strategies and even reduce motivation for use. Therefore, designing feedback mechanisms that align with cognitive principles, enabling users to understand the system state and gradually establish stable operational patterns, constitutes a critical issue in BCI system design.

The establishment and maintenance of human–trust relationships directly affect the long-term effectiveness of BCI systems [71]. When the system frequently exhibits misrecognition or response delays, users’ judgments and expectations regarding system capabilities shift accordingly, further influencing their operational behavior and continued usage intention. Consequently, enhancing the transparency and comprehensibility of the interaction process, so that users can form reasonable expectations and build stable trust, is an essential prerequisite for achieving long-term brain–computer synergy.

Of course, relying solely on improving decoding accuracy is insufficient to resolve all problems. Given the inherent uncertainty of brain signals, well-designed fault-tolerance mechanisms remain a critical safeguard for system usability and safety [72]. Effective fault-tolerance design should not only reduce the risk of erroneous operations, but also minimize the cognitive burden and psychological stress associated with error correction. For instance, by incorporating contextual information, task context, and dynamic interaction strategies, BCI systems can enhance overall robustness and user acceptance while maintaining interaction efficiency.

Beyond trust and fault tolerance, BCIs also present a set of unique human factors challenges inherent to the brain–computer interaction mechanism itself. Some users may disengage from BCI use due to difficulties in developing stable, decodable brain signal patterns or a lack of understanding of the boundaries of BCI capabilities. Accordingly, BCI competence, user screening, and expectation management have emerged as critical topics [73].

Conventional BCIs typically require lengthy individual calibration. The substantial time cost and user burden have driven advances in reduced-calibration, zero-calibration, plug-and-play, and transfer learning approaches. ErrP captures users’ implicit awareness of system errors, providing neural-level support for human–machine hybrid error correction and fault-tolerant design.

Meanwhile, disparities in cognitive, motor, and sensory capacities among older adults, users with disabilities, and those with severe communication impairments position accessibility and universal design as core priorities for BCI deployment. Furthermore, autonomy, sense of agency, and sense of embodiment during brain-controlled operation relate to users’ perceptions of control, action attribution, and the boundaries of system automation, and shape whether users perceive external devices as an extension of their own capabilities.

These issues embody the distinctiveness of Human Factors Engineering for BCIs across the dimensions of individual differences, interaction burden, fault tolerance mechanisms, accessibility, and human–machine relationships, and intersect with research directions including individual adaptation, co-adaptation, and contextual adaptation.

The above issues indicate that human factors optimization in BCI interaction depends not only on improvements in signal-processing performance, but also on users’ understanding of system behavior, the establishment of trust relationships, and the refinement of fault-tolerance capabilities. Cognitive adaptation, trust construction, and fault-tolerant synergy together constitute important human factors considerations in BCI system design, and further relate to the differences and commonalities in human factors requirements across various application scenarios.

### 3.3. General Laws of Scenario-Based Human Factors Adaptation

As BCI technology progressively enters application domains such as medical rehabilitation, industrial control, education and training, and immersive interaction, the differences in human factors requirements across scenarios become increasingly prominent. An important question thus arises: What factors underlie these differences, and are there cross-scenario human factors design principles and evaluation frameworks that can be universally applied?

From existing research, human factors requirements are first influenced by the specific task context. Different application environments exhibit marked variations in task objectives, user characteristics, risk levels, and operational conditions, and consequently, the human factors priorities differ accordingly [74].

In medical rehabilitation scenarios (e.g., stroke-related upper limb rehabilitation), users are mostly patients with motor function impairments. The human factors focus lies in long-term training adherence, training intensity and fatigue tolerance, neural adaptation during motor function remodeling, and objective evaluation of rehabilitation outcomes [75,76,77].

In assistive communication and environmental control scenarios, users may be patients with severe motor disorders such as amyotrophic lateral sclerosis (ALS), with extremely limited available interaction pathways. The human factors priority is to achieve highly reliable selection, strong fault tolerance, and comfort during prolonged use with minimum cognitive and motor burden, while preserving users’ autonomy and dignity [78,79].

In transportation and industrial control scenarios, users are predominantly healthy operators, and systems typically center on passive monitoring of attention, fatigue, and distraction. The human factors emphasis is placed on alarm thresholds, the balance between false alarms and missed alarms, emergency takeover time, and operational safety [80].

In education, gaming, and immersive environments, users attach greater importance to immersion, engagement, and learning motivation. The human factors focus is on striking a balance between immersive experience, cognitive load, and visual fatigue, as well as the synergistic design of VR feedback and neurofeedback [81].

A cross-scenario comparison reveals that comfort, safety, cognitive load control, interaction transparency, and trust represent relatively universal requirements, while specific indicator thresholds, feedback modalities, and evaluation weights vary across scenarios.

However, scenario differences do not imply a lack of commonalities. Regardless of the specific application, factors such as comfort, safety, cognitive load control, interaction transparency, and user trust consistently influence the practical usability and long-term acceptance of BCIs [74]. These shared concerns form an important foundation for cross-scenario human factors design and offer the possibility of establishing a unified evaluation framework.

How to synthesize generalizable human factors design principles while preserving scenario-specific distinctions remains a question worthy of further discussion [74]. In the future, it may be necessary to establish more unified core metrics and evaluation frameworks, so that human factors designs across different application environments can both address specific needs and maintain consistency in fundamental principles, thereby enhancing the comparability and transferability of research findings.

Scenario-based adaptation reflects the strong dependence of BCI systems on specific task environments, yet different scenarios are not entirely disconnected. Issues such as comfort, safety, cognitive load, and user trust still constitute the common human factors foundation shared by many applications. Further synthesis of these general laws will not only help refine the theoretical framework of BCI human factors engineering, but also strengthen the generalizability and transferability of relevant design methods.

### 3.4. Human Factors Ethics, Neural Data Privacy, and User Rights and Interests

As BCIs expand into clinical, assistive, and high-risk application scenarios, ethical and rights issues deeply intertwined with human factors have increasingly become an integral part of system design and user research [82,83]. Brain signals are highly individual-specific, and their acquisition and analysis may involve information related to emotion, attention, cognitive states, and even personal identity. Accordingly, full consideration must be given to informed consent, purpose limitation, data minimization, encryption safeguards, and access control throughout the collection, storage, transmission, and sharing of neural data. Applications involving personal identification or state inference should also be transparently disclosed to users [84,85].

As BCI systems gain enhanced adaptive and autonomous execution capabilities, the scope of human oversight, veto, and takeover authority, as well as the boundaries of liability in cases of system failure, also need to be clearly defined at the design stage. Meanwhile, users should be fully informed about the practical capability boundaries of BCIs, the potential phenomenon of “BCI illiteracy”, and the uncertainties inherent in long-term use, so as to form reasonable expectations and avoid overpromising.

Technology deployment should also address disparities in access to use, operational burden, and degree of benefit among users of different ages, disability statuses, and socioeconomic conditions, to avoid creating new inequities driven by technical barriers [86]. These ethical and rights issues interact with user trust, acceptance, and willingness for long-term use, such that human factors research on BCIs must go beyond addressing whether systems are “usable” and “effective”, and further examine factors including user rights, sense of control, level of informedness, and equitable access to benefits.

## 4. Methodological Framework of BCI Human Factors Engineering

The methodological framework of BCI human factors engineering builds upon traditional human factors research; however, owing to the direct involvement of brain signals in the interaction process, its research methods further integrate neural measurement, closed-loop experimentation, and cognitive analysis. Compared with traditional human factors studies, BCI human factors research requires not only observation of users’ behavioral performance, but also attention to brain-state changes, learning processes, and adaptation characteristics during long-term use.

Consequently, BCI human factors research typically combines information from neural, cognitive, and behavioral levels simultaneously to conduct a comprehensive analysis of the brain–computer interaction process. This paper summarizes the relevant methods into three categories: multimodal measurement and user modeling, human factors validation under closed-loop interaction conditions, and multi-dimensional evaluation methods tailored for BCI systems.

### 4.1. Multimodal Measurement and User Modeling Methods for Neurocognitive Characteristics

Measuring users’ neural activity and cognitive states is the first issue that must be addressed in conducting BCI human factors research. Since brain signals not only reflect control intentions but are also influenced by factors such as attention, fatigue, and task stress, relying solely on behavioral outcomes or peripheral physiological indicators often fails to fully characterize users’ actual states during interaction. Therefore, multimodal measurement has gradually become a commonly adopted approach in BCI human factors research [87].

In specific studies, neural signals, behavioral performance, and peripheral physiological information are typically combined and analyzed together to observe changes in user states and their effects on the interaction process. The cross-validation of multi-source information facilitates a more comprehensive understanding of the relationship between cognitive processes and behavioral performance, and provides richer evidence for interaction design and system optimization [74]. For learning effects and adaptation processes that emerge during long-term use, such methods also support continuous monitoring and dynamic analysis.

In terms of user modeling, BCI human factors research is increasingly emphasizing information at the neural-feature level. Beyond behavioral experience and task context, brain activity patterns, users’ cognitive strategies, and the temporal characteristics of state changes are progressively being incorporated into user models [88]. Based on such information, the system can make appropriate adjustments to training protocols, interaction parameters, and feedback modalities, thereby better accommodating different users and their state fluctuations.

Artificial intelligence (AI) is transforming how human factors research on BCIs addresses user states, individual differences, and long-term adaptation issues [89]. Conventional approaches typically rely on handcrafted feature design and limited individual calibration. By contrast, machine learning and deep learning can learn high-dimensional representations from heterogeneous data sources including electroencephalographic (EEG) signals, eye movements, peripheral physiological measures, and behavioral data, providing more dynamic analytical tools for the identification of implicit states such as cognitive load, fatigue, and attention.

This data-driven capability also enables individual differences among users to shift from “variability to be controlled” to modelable information. Through metric learning, clustering, and individualized models, differences in neural representations across users can be identified, supporting differentiated configuration of paradigms and parameters. In cross-session applications, transfer learning, domain adaptation, and online learning further enable models to adapt to new users and states using existing data, reducing the impact of repeated calibration on the usage process.

Meanwhile, deep network modeling of ERD/ERS patterns, functional connectivity, and temporal dynamics opens up new possibilities for predicting user states and enabling proactive system adjustment. However, data-driven approaches do not automatically resolve human factors issues. Model interpretability, reliability under conditions of small sample sizes and distribution shift, as well as users’ understanding, trust, and oversight of algorithmic decisions, can all shape real-world human–machine collaboration.

Accordingly, the significance of AI in BCI human factors research lies not only in improving decoding or state recognition performance, but more importantly in driving the evolution of BCIs from static, uniform interaction paradigms to intelligent interactive systems that can perceive user states and continuously adapt to individual differences [17].

These methods enable researchers to observe not only final behavioral outcomes, but also a more complete linkage among neural activity, cognitive processes, and external manifestations, thereby offering novel observational perspectives for understanding adaptation processes during long-term use.

### 4.2. Human Factors Validation Methods Under Closed-Loop Interaction Conditions

Owing to the continuous feedback and online adjustment processes inherent in BCI systems, human factors research in BCIs often necessitates validation under closed-loop conditions, rather than relying solely on offline experiments or single-session task tests. Compared with traditional experimental paradigms, closed-loop validation is more conducive to observing processes such as user learning, strategy adjustment, and long-term adaptation, and has thus gradually become an important approach in BCI human factors research.

To enhance the consistency between experimental results and real-world applications, researchers often employ virtual reality, digital twin environments, or scenario-based experimental platforms to simulate complex tasks such as medical rehabilitation and industrial control under controllable conditions. This approach not only ensures the reproducibility of experimental conditions, but also facilitates continuous observation of users’ behavioral adjustments and cognitive changes during extended interaction, thereby improving the ecological validity of the research.

Consequently, closed-loop experiments typically do not merely record final task outcomes, but also concurrently examine the evolution of user states throughout the learning process, including cognitive load, strategic adjustments, and adaptation speed [90]. Through continuous observation of the interactive dynamics between the user and the system, a more comprehensive analysis of brain–computer coordination characteristics under long-term usage conditions can be achieved [91].

In contrast to offline evaluation, closed-loop validation places greater emphasis on the interaction process itself rather than on single-session task results. Researchers are concerned not only with system performance, but also with how users learn, how they adjust their strategies, and how the mutual adaptation between the two unfolds over extended use—a distinguishing feature that sets BCI human factors research apart from conventional usability testing.

### 4.3. Multi-Dimensional Human Factors Evaluation Methods for BCIs

Human factors evaluation for BCI systems is no longer confined to usability metrics. Beyond task completion and interaction efficiency, users’ cognitive load, usage experience, and long-term adaptation processes also significantly affect the practical application outcomes of the system. Consequently, BCI human factors evaluation often requires simultaneous consideration of multiple dimensions, including behavioral performance, user experience, and neural states [74]. Currently, relevant research can be broadly categorized into dimensions including effectiveness, efficiency, cognitive load, usability, satisfaction, trust, acceptance, comfort, and neural adaptability, as outlined in Table 4.

In the past, research has placed greater emphasis on accuracy and information transfer rate. However, as BCIs progressively enter real-world application environments, increasing attention is being paid to whether users can maintain stable usage over the long-term and whether they are willing to continue accepting the system. Correspondingly, users’ neural states and their adaptation processes are becoming important components of the human factors evaluation framework.

Related studies typically combine brain activity features, cognitive load indicators, and behavioral performance to analyze changes in user states and their relationship with system performance. These neural-level data contribute to understanding the long-term evolutionary characteristics of the brain–computer co-adaptation process, and also provide a basis for personalized design and subsequent optimization.

Among these dimensions, neural adaptability is a unique dimension that distinguishes human factors evaluation for BCIs from usability evaluation. It refers to the measurable adaptive changes in brain activity patterns and brain network connectivity occurring through learning and co-adaptation during users’ repeated BCI use, as well as the degree of co-evolution between these neural changes and system performance.

Currently, BCI human factors evaluation is no longer merely a measurement of task outcomes and operational efficiency; user states, long-term experience, and the brain–computer adaptation process is increasingly becoming integral components of evaluation. How to reconcile behavioral indicators, subjective assessments, and neural information, and how to establish an evaluation framework that accommodates the requirements of different application scenarios, remain open questions that warrant continued exploration in this field.

### 4.4. Application Path of the Methodological System in the Full BCI Development Lifecycle

The previous sections constructed the methodological system of Human Factors Engineering for BCIs across three dimensions: measurement, validation, and evaluation. It should be noted that multimodal measurement and user modeling, closed-loop validation, and multidimensional evaluation are not independent research instruments. Instead, they should be embedded throughout the full BCI development lifecycle—from requirements definition, prototype development, and testing and validation to long-term iteration—to form an engineering-oriented development pathway. Only by aligning these methods with specific development phases can human factors research be translated from laboratory findings into actionable specifications that guide real-world BCI design, and thereby tangibly improve the quality of human–machine interaction.

Following a user-centered design process and incorporating the technical characteristics of BCI systems, the development process can be divided into four typical phases: requirements definition and user modeling, interaction prototyping and algorithm iteration, product testing and scenario validation, and long-term deployment and iterative optimization. The human factors objectives, core methods, and deliverables vary across phases, yet they form a closed loop through data and feedback, as outlined in Table 5.

As shown in Table 5, multimodal measurement methods function primarily in the early development phase to establish user baselines and individualized models. Closed-loop validation methods are concentrated in the prototype iteration phase, where they verify interaction logic and feedback mechanisms. Multidimensional evaluation methods span the product testing and long-term deployment phases, and feed evaluation results back into requirements definition and prototype iteration to form a cross-phase feedback loop.

In the requirements definition and user modeling phase, the core role of multimodal measurement is to translate abstract user differences into actionable design inputs. Development teams should conduct systematic multimodal baseline measurements on target user groups, covering neural activity characteristics (e.g., EEG rhythms, event-related potential response patterns), cognitive abilities (e.g., attentional stability, working memory capacity), peripheral physiological features (e.g., heart rate variability, eye movement patterns), and behavioral performance (e.g., learning speed, strategy preferences). On this basis, users are stratified into groups with similar neurocognitive characteristics through clustering, metric learning, or individualized modeling, which informs the determination of interaction paradigms, parameter ranges, and training pathways. For example, for MI-BCIs, users can be classified into highly decodable, moderately decodable, and poorly decodable groups (i.e., potential “BCI illiteracy” users) based on the separability strength of ERD/ERS in the somatosensory cortex, with differentiated paradigm selection and training schemes applied to each group. For SSVEP-BCIs, appropriate flickering frequency combinations and stimulus durations can be recommended for different users based on the response properties of the occipital visual cortex and susceptibility to visual fatigue. This user stratification, grounded in multimodal measurement, avoids the design tendency of applying a one-size-fits-all paradigm from the outset of development and forms the foundation for improving BCI usability and long-term adherence. Meanwhile, environmental constraints in the target scenario (e.g., illumination, noise, wear conditions) and task characteristics (e.g., risk level, operation frequency) should also be measured concurrently. Integrating user neurocognitive characteristics with scenario constraints yields a complete human factors requirement specification, which provides the basis for subsequent prototype design.

When BCI systems enter the interaction prototyping and algorithm iteration phase, closed-loop validation methods become the core tool. Unlike offline data acquisition and single-session task testing, closed-loop validation emphasizes continuous observation of the dynamic relationships among users’ learning processes, strategy adjustments, and system feedback under online system operation conditions, and iteratively optimizes interaction design accordingly. Implementation proceeds in three stages. First, offline accuracy verification: the baseline performance of decoding algorithms is tested on calibration datasets to ensure the system possesses basic control capability. Second, closed-loop experience validation: closed-loop tasks simulating real-use environments are constructed on virtual reality, digital twin, or scenario-based experimental platforms. The focus is on observing users’ learning curves, error patterns, cognitive load changes, and feedback responsiveness, rather than solely on offline accuracy. For instance, in feedback mechanism design, closed-loop experiments compare the effects of different feedback delays, channel allocations, and redundant coding schemes on users’ learning speed and control continuity. In fault-tolerant design, hybrid error correction strategies are evaluated against error operation rates and correction burden by combining ErrP detection with users’ overt behaviors. Third, scenario stability validation: prototypes are deployed in near-real application environments to verify system stability under conditions of interference, fatigue, and prolonged use, and to record users’ interaction performance under real task constraints. Through this three-level closed-loop validation, development teams can identify human factors issues such as improper feedback timing, excessive cognitive load, and failed fault tolerance strategies at the prototype stage, and implement rapid iterative corrections to prevent design flaws from carrying over into subsequent product testing phases. This process also serves as empirical verification of the brain–computer co-adaptation mechanism: the system adjusts control parameters based on user states, while users adjust cognitive strategies based on system feedback, forming bidirectional optimization within closed-loop experiments.

In the product testing and long-term deployment phases, multidimensional evaluation methods undertake the function of comprehensively assessing the quality of human–machine interaction. As previously described, human factors evaluation of BCIs covers multiple dimensions including effectiveness, efficiency, cognitive load, usability, satisfaction, trust, acceptance, comfort, and neural adaptability. In engineering practice, the key is to organize these dimensions into a phased evaluation matrix and define the translation pathway from evaluation results to design improvements. In the product testing phase, evaluation priorities should be determined according to BCI type and scenario characteristics. For medical rehabilitation BCIs, focus should be placed on training effectiveness, cognitive load, adherence, and degree of motor function remodeling. For assistive communication BCIs, selection reliability, comfort during prolonged use, and user autonomy should be prioritized. For consumer control BCIs, a balance must be struck among operation efficiency, visual fatigue, and usage intention. Upon completion of evaluation, a structured human factors issue list should be output, specifying the direction of design improvement for each issue. For example, when cognitive load indicators exceed thresholds, priority should be given to simplifying feedback channels, reducing information redundancy, or adjusting task rhythm. When trust is low, system state transparency and error explanation should be enhanced. When neural adaptability improves slowly, training intensity and neurofeedback strategies should be adjusted. In the long-term deployment phase, multidimensional evaluation should be transformed into a continuous tracking mechanism. By regularly collecting multimodal data (neural states, behavioral performance, subjective feedback), the evolution of users’ neural patterns, fatigue accumulation patterns, and adherence changes can be monitored over weeks or even months of use. These long-term data serve two purposes: on one hand, they update individualized user models to enable the system to continuously adapt to users’ neuroplastic changes; on the other hand, they identify degradation issues arising from long-term use (e.g., declining signal stability, fading usage interest) to inform product feature iteration. This forms a long-term closed loop.

To further illustrate the engineering implications of the above pathway, the concrete application of the methodological system is outlined below for two typical BCI scenarios. In the development of a stroke upper limb rehabilitation BCI, the requirements definition phase conducts multimodal baseline measurements on admitted patients, including resting EEG, motor function assessment, cognitive status, and fatigue tolerance, based on which user stratification is performed and personalized training intensity and paradigm selection are formulated. In the prototype iteration phase, closed-loop training experiments are conducted based on VR rehabilitation scenarios, with simultaneous collection of EEG, limb movement data, and subjective workload feedback to optimize the visual-tactile multimodal feedback scheme and adaptive training difficulty rules. The focus is on validating patients’ learning curves, ErrP-driven error correction strategies, and real-time detection and intervention of fatigue states during training. In the product testing and long-term follow-up phase, training effectiveness, cognitive load, training adherence, and degree of motor function remodeling are evaluated in stages, and changes in neural adaptability are continuously monitored over weeks to months of follow-up. Training schemes and neurofeedback strategies are adjusted accordingly, with the ultimate goal of improving rehabilitation outcomes and long-term training adherence. In the development of a consumer-grade SSVEP BCI for smart home control, the requirements definition phase identifies visual fatigue thresholds, attentional resource allocation characteristics, and cognitive load levels across different user groups through multimodal measurement, which informs optimization of flickering frequency combinations, stimulus duration, and interface layout. In the prototype iteration phase, closed-loop usability testing is conducted in a simulated home environment, focusing on fatigue changes, false operation rates, and feedback response experience over 30 min of continuous use. Prompt modalities, error feedback timing, and automatic pause mechanisms for non-critical commands are continuously optimized. In the product testing and long-term deployment phase, comprehensive evaluation is performed across dimensions such as operation accuracy, response efficiency, visual comfort, usage intention, and trust. Long-term tracking monitors users’ visual fatigue accumulation and usage frequency changes to balance technical performance and user experience, and enhance product acceptance and willingness for sustained use.

The above application pathway demonstrates that multimodal measurement and user modeling, closed-loop validation, and multidimensional evaluation are not isolated research tools, but rather a human factors-driven method chain that can be embedded in the full BCI development lifecycle. Multimodal measurement in the requirements phase translates user neurocognitive differences into personalized design inputs. Closed-loop validation in the prototype phase verifies and optimizes feedback and fault tolerance mechanisms under realistic interaction conditions. Multidimensional evaluation in the testing and long-term deployment phases provides continuous feedback to drive system iteration and optimization. The core value of this pathway lies in translating the human-centered design philosophy from abstract principles into actionable engineering specifications, enabling BCI systems to not only be technically functional, but also achieve low workload, high trust, and sustainable human–machine collaboration in real-world use.

## 5. Disciplinary Value and Future Directions

### 5.1. Core Rationale for the Emerging Interdisciplinary Research Direction

BCI technology is progressively moving beyond the laboratory and entering real-world application environments such as medical rehabilitation, industrial control, and immersive interaction. As application scenarios continue to expand, many theories and methods from traditional human factors engineering remain foundational, yet they do not fully cover certain mechanisms that become prominent when central neural activity serves as the control channel and adaptation is bidirectional and long-term. In this process, human factors research centered on BCIs has accumulated steadily, gradually giving rise to relatively clear research boundaries, core questions, and methodological pathways, and showing a trend toward an emerging interdisciplinary research direction.

The preceding analysis demonstrates that BCI human factors engineering exhibits characteristics that distinguish it from existing human factors research in terms of research objects, core questions, and methodological frameworks. It not only addresses interaction processes at the behavioral level, but also incorporates neural activity and cognitive states into the analytical framework of human–system coordination. Its core questions encompass individual differences, brain–computer co-adaptation, trust formation, and scenario-based adaptation. In terms of research methods, it emphasizes the integration of multimodal measurement, closed-loop validation, and longitudinal evaluation. These features collectively constitute a relatively distinct problem domain and research paradigm, providing a foundation for its development as an emerging research direction.

More importantly, the development of BCI human factors engineering can also drive the extension of the human factors research perspective itself. Human–computer interaction has traditionally emphasized overt interaction while also addressing implicit and physiological input, whereas BCIs further incorporate neural activity and cognitive states into the processes of system design, evaluation, and optimization. The expansion of research objects—from the relationship between behavior and system to the triadic relationship among neural, cognitive, and systemic factors—does not represent a replacement of existing human factors engineering, but rather a further extension and deepening of the human-centered philosophy under new interaction conditions.

Therefore, BCI human factors engineering is not merely a simple application of traditional human factors methods to the BCI domain. As its research objects, core questions, and methodological systems become increasingly well defined, it is gradually developing a problem domain and research paradigm with its own distinct characteristics. For Human Factors Engineering for BCIs to develop into a widely recognized emerging interdisciplinary research direction, continuous improvement is required across its theoretical framework, methodological standards, and academic ecosystem. For instance, it is necessary to gradually establish actionable and generalizable research paradigms and measurement standards, build a core evaluation indicator system and shared data resources that support cross-study comparisons, and accumulate systematic evidence through long-term, replicable validation across different BCI paradigms and real-world application scenarios. Meanwhile, efforts should also be made to promote the development of relevant academic communities, education and training systems, and professional communication platforms. Whether it will eventually become established as an emerging interdisciplinary research direction still depends on further empirical validation and theoretical accumulation; nevertheless, its trajectory as an important emerging direction within human factors engineering has become increasingly clear.

### 5.2. Future Key Research Directions

Although BCI human factors engineering has established an initial research framework, many issues remain to be further explored as BCIs progressively enter long-term usage and complex application environments. From the perspective of current development trends, future research priorities are likely to concentrate more on trustworthy collaboration and cross-scenario design.

Trustworthy human–machine synergy mechanisms equally warrant attention. Given the inherent decoding errors and system uncertainties in brain–computer interaction, how users interpret system behavior, form reasonable expectations, and maintain long-term trust will directly affect the practical efficacy of the technology. Issues such as privacy protection of brain signals, user autonomy, and boundaries of responsibility not only involve technical implementation but also relate to user acceptance and long-term adoption, and should therefore remain persistent focuses of future human factors research.

As application scenarios continue to expand, cross-scenario human factors design principles and evaluation standards are becoming increasingly important. Considerable discrepancies still exist across different domains in terms of experimental paradigms, evaluation metrics, and long-term usability studies, limiting the comparability and transferability of research findings. Establishing a more unified human factors evaluation framework may serve as a critical foundation for promoting engineering applications and standardization efforts.

From a longer-term perspective, the development of BCIs is no longer merely a matter of improving accuracy or control capability. Whether users can maintain stable system use over extended periods, and whether they are willing to trust and continue accepting this novel interaction modality, equally determine the ultimate application value of the technology. These issues also open up new research avenues for the future development of BCI human factors engineering.

Meanwhile, a balanced and objective perspective should be adopted regarding the boundaries of the role of Human Factors Engineering for BCIs. Human factors optimization can effectively mitigate barriers to real-world deployment, including user adaptation difficulties, heavy calibration and training burdens, poor long-term adherence, and inadequate user experience and acceptance, making systems more usable and more practical within existing technological constraints. However, it cannot replace breakthroughs in foundational technologies such as acquisition hardware, neural decoding algorithms, and the biocompatibility of implant materials, and yields only limited improvements to hard constraints such as signal bandwidth and accuracy.

The large-scale, real-world deployment of BCIs is the outcome of the collaborative advancement of human factors engineering and technical engineering. One-sided emphasis on either dimension alone is unrealistic.

## 6. Conclusions

Focusing on the human factors bottlenecks hindering the large-scale deployment of BCIs, this paper reviews the research scope, core scientific questions, and methodological framework of BCI human factors engineering. The implicit nature of BCI input, dynamic fluctuations in neural states, and the bidirectional brain–computer co-adaptation characteristics are aspects that existing human factors frameworks address only partially, which constitutes the fundamental rationale for establishing this dedicated interdisciplinary research direction. This paper identifies four categories of core research objects in the field and clarifies its disciplinary boundaries with existing human factors engineering and BCI engineering technology. On this basis, three categories of core scientific questions are synthesized, and a corresponding methodological framework is constructed, encompassing multimodal measurement and user modeling, closed-loop interaction validation, and multi-dimensional human factors evaluation.

The essence of BCI human factors engineering lies in the synergistic adaptation among neural activity, cognitive processes, and system design, rather than a mere application of traditional human factors methods to a new domain. The related research can not only provide theoretical support for human-centered BCI system design, but also help enhance long-term system usability and practical application value, while further extending the research boundaries of the human factors discipline.

Meanwhile, artificial intelligence and multimodal sensing provide new tools for personalized modeling and temporal adaptation, while ethical issues such as neural data privacy and user autonomy should be considered in parallel with technological development. This also represents a key direction for continuous deepening within Human Factors Engineering for BCIs going forward.

## Figures and Tables

**Table 1:** Seven typical constituent elements of BCI systems.

No.	Typical Constituent Elements of BCI Systems
1	User and their brain
2	BCI paradigm and neural encoding module
3	Brain-signal acquisition module
4	Online real-time intent decoding module
5	Output mapping and peripheral-device control module
6	Communication and control peripherals
7	Sensory feedback combined with neurofeedback module

**Table 2:** Human factors characteristics of BCI interaction.

No.	Human Factors Characteristic	Specific Manifestation	Resulting Human Factors Issues
1	Implicitness of input	Central neural activity, rather than limb behavior, serves as input	User intent is not directly observable; state perception and interaction transparency are insufficient
2	Brain–computer co-adaptation	User learning and system updates proceed concurrently	Difficulties in long-term dynamic adaptation and collaborative optimization
3	Dynamic nature of neural states	Fatigue, attention, and emotion continuously affect brain signals	Limited system stability and real-time adaptation capability
4	High cognitive load	Brain-controlled tasks depend on sustained attention and mental effort	Increased cognitive fatigue and operational burden
5	Significant individual differences	Marked variations in neural patterns and cognitive strategies across users	Uniform paradigms cannot satisfy personalized requirements
6	Dependence on long-term training	Skill acquisition relies on prolonged learning and neuroplasticity	Long-term compliance and sustained usage experience become critical

**Table 3:** Research objects of related fields and their relationships with HFE for BCIs.

Related Field	Research Object	Relationship with HFE for BCIs
Human Factors Engineering/Cognitive Ergonomics	Humans in work systems; workload, attention, situational awareness, trust, automation, safety, and comfort [10]	Provides the parent theories and methodologies (workload, trust, adaptive automation, etc.); HFE for BCIs represents its specialized extension to the BCI pathway [40]
Neuroergonomics	Brain and neural mechanisms under real working conditions; explaining workload and performance through brain measurements [41]	Provides the basis for neural measurement and mechanisms; brain activity in neuroergonomics is mostly treated as an object of observation, while HFE for BCIs further treats brain activity as control signals and investigates closed-loop co-adaptation [42]
Human–Computer Interaction/Usability Engineering	Interaction processes between users and systems, usability, and user experience [43]	Provides usability engineering and UX evaluation methods; HFE for BCIs supplements this framework with new dimensions such as neural adaptation and implicit load [44]
BCI Engineering Technology	Brain signal acquisition, feature decoding, algorithms, and hardware systems, pursuing accuracy, speed, and stability [11]	Provides technical tools and platforms; HFE for BCIs focuses on the overall human-system performance, and the two are complementary rather than substitutive [45]
User-Centered BCI Design	BCI design processes and usability evaluation oriented toward end-user needs [46]	Constitutes the concrete practice of HFE for BCI concepts in the design workflow; HFE for BCIs provides it with theoretical foundations, methodologies, and evaluation systems [46]
Co-adaptive/Neuroadaptive Technology	Closed-loop adaptation between users and systems based on neural or physiological states; real-time adjustment of task difficulty, automation level, and interface parameters [47]	Provides core mechanisms and methods for brain–computer co-adaptation, adaptive automation, and neuroadaptive interaction; HFE for BCIs extends these to BCI-specific contexts by integrating long-term co-adaptation, user experience, and neuroadaptive technology [47,48]

**Table 4:** Main dimensions and typical indicators of human factors evaluation for BCIs.

Evaluation Dimension	Typical Indicators	Key Focus
Effectiveness	Accuracy, error rate, decoding stability [12]	Task completion capability and control reliability
Efficiency	ITR (information transfer rate), response time, training cycle, learning cost [11,92]	Interaction speed and long-term use efficiency
Cognitive load	NASA-TLX, subjective workload rating, EEG/peripheral physiological workload indicators [60,80,93]	Mental resource consumption, fatigue, and attentional occupation
Usability	SUS, PSSUQ, task operation issues [94,95]	Perceived ease of use and learnability
Satisfaction	QUEST and other satisfaction questionnaires, user interviews [74,96,97]	Usage satisfaction and expectation fulfillment
Trust	Human–machine trust scales, dependency/compliance behaviors, trust calibration [98,99]	Appropriate and rational trust under false alarms and missed alarms
Acceptance	TAM/UTAUT scales, usage intention, continued use [100,101]	Technology adoption and long-term usage
Comfort	Wear comfort scales, local pain/fatigue reports, tolerable wear duration [102]	Hardware wear burden and long-term use experience
Neural adaptability	Functional connectivity and graph theory metrics, neuroplasticity indicators, cross-session signal stability [61,91,103,104]	Long-term neural adaptation and brain–computer co-adaptation processes

**Table 5:** Mapping of human factors methods in the full BCI development lifecycle.

Development Phase	Core Human Factors Objective	Primary Applied Methods	Key Deliverables
Requirements Definition and User Modeling	Identify neurocognitive characteristics and usage constraints of target users	Multimodal measurement and user modeling	User stratification models, personalized requirement specifications, paradigm selection criteria
Interaction Prototyping and Algorithm Iteration	Validate the effectiveness and learning curves of closed-loop interaction	Closed-loop validation methods	Feedback mechanism schemes, fault tolerance strategies, adaptive control parameters
Product Testing and Scenario Validation	Comprehensively evaluate system usability, workload, and acceptance	Multidimensional evaluation methods	Human factors issue list, design improvement priorities, scenario adaptation schemes
Long-Term Deployment and Iterative Optimization	Monitor neural adaptation and adherence during long-term use	Continuous tracking via multimodal measurement and multidimensional evaluation	Long-term training programs, personalized iteration strategies, co-adaptation models

## Data Availability

No new data were created or analyzed in this study. Data sharing is not applicable to this article.
